# Neglected Joint Infection Occurring Following Intra‐Articular Injection and Colon Perforation: A Case Report

**DOI:** 10.1155/cro/1431586

**Published:** 2026-04-25

**Authors:** Fabio Massimo Abenavoli, Omar Tujjar, Andrea Alfonso

**Affiliations:** ^1^ AVOCA Clinic, Kilmacanogue, Co Wicklow, Dublin, Ireland; ^2^ National Orthopaedic Hospital Cappagh, Dublin, Ireland; ^3^ Department of Anaesthesia, Cambridge University Hospital NHS Foundation Trust, Cambridge, UK, nhs.uk

**Keywords:** antimicrobial stewardship, gut microbiota dysbiosis, septic arthritis of the knee, source control, *Streptococcus anginosus*

## Abstract

**Introduction:**

Septic arthritis is an uncommon but severe complication of intra‐articular procedures and is associated with significant morbidity and mortality when diagnosis or source control is delayed. Large cohort data have demonstrated adverse joint and systemic outcomes even when surgical washout is performed.

**Case Presentation:**

We report the case of a 64‐year‐old woman with Type 2 diabetes mellitus who developed septic arthritis of the knee 2 days after intra‐articular hyaluronic acid injection, a procedure generally considered safe but known to carry a small risk of infection. Synovial fluid cultures identified *Streptococcus anginosus* and subsequently *Streptococcus gordonii*, organisms recognised for their propensity to cause invasive and disseminated infections. Despite prolonged hospitalisation and exposure to multiple sequential broad‐spectrum antibiotic regimens, the infected joint was not surgically drained. During treatment, the patient developed persistent diarrhoea, abdominal pain and systemic inflammatory features. Repeated testing for *Clostridioides difficile* was negative, despite recognised associations between antibiotic exposure, acid‐suppressive therapy and enteric complications. Computed tomography later demonstrated intestinal dilatation and ultimately pneumoperitoneum. Emergency bowel resection was performed, but the patient died shortly thereafter from multiple organ failure. Histopathological examination revealed extensive inflammatory ulceration of the colon.

**Conclusions:**

This case illustrates a complex and fatal clinical course occurring in the context of persistent septic arthritis managed without joint drainage and prolonged antimicrobial exposure. Although causality cannot be established from a single report, the case reinforces the importance of timely source control in native joint septic arthritis, highlights the consequences of prolonged empirical antimicrobial therapy in the absence of adequate surgical debridement and underscores the need for early multidisciplinary reassessment when gastrointestinal symptoms arise during prolonged hospitalisation.

## 1. Introduction

Intra‐articular hyaluronic acid injections are widely used in the management of symptomatic knee osteoarthritis and are generally regarded as safe. Reported adverse events are typically mild and self‐limiting; however, septic arthritis remains a recognised, though infrequent, complication [1–3]. When septic arthritis occurs, it represents a medical emergency associated with significant morbidity and mortality [4].

Septic arthritis of a native joint requires prompt diagnosis, appropriate antimicrobial therapy and, in most cases, early joint drainage to achieve adequate source control [5, 6]. Failure to control the infectious focus may result in persistent inflammation, prolonged hospitalisation and repeated exposure to broad‐spectrum antibiotics. Prolonged and combined antimicrobial therapy has been associated with disruption of intestinal microbial homeostasis and increased risk of inflammatory bowel conditions [7–10]. The concomitant use of proton pump inhibitors and corticosteroids may further exacerbate this risk by altering gastric acidity and mucosal immune defences [11]. Although a single case cannot establish causality, detailed reporting may help identify clinically relevant associations and system‐level vulnerabilities.

## 2. Patient Information

A 64‐year‐old woman with a known diagnosis of Type 2 diabetes mellitus underwent intra‐articular hyaluronic acid injection of the knee for the treatment of a painful joint condition presumed to be degenerative in nature. The patient was physically active and had previously worked as a physiotherapist. At the time of presentation, she was involved in regular physically demanding activities related to providing care for her husband, who had a severe physical disability. Body mass index was over 25. There was no tobacco or alcohol use and no relevant psychosocial or lifestyle factors affecting the patient.

Her medical history was notable for Type 2 diabetes mellitus, a recognised risk factor for septic arthritis and adverse infectious outcomes [12]. The patient was moderately obese and had a history of knee arthralgia with functional limitation, for which she had received several intra‐articular hyaluronic acid injections in previous years. Additional comorbidities included arterial hypertension, managed with medical therapy, and hypercholesterolaemia, treated with statins. There was no documented history of chronic kidney disease, gastrointestinal disease, inflammatory bowel disease, inflammatory joint disease or conditions associated with immunosuppression.

## 3. Clinical Findings

Two days after the intra‐articular injection, the patient developed acute knee pain, swelling, functional limitation and fever, a presentation consistent with septic arthritis as described in previous series of postinjection infections [1–3]. On presentation to the emergency department, the working diagnosis was septic arthritis of the left knee following intra‐articular hyaluronic acid injection. Physical examination revealed oedema of the left knee, marked tenderness on palpation and significant limitation of flexion and extension. Local signs of inflammation were present. Detailed systemic vital parameters at presentation were not documented in the available records.

During hospitalisation, the patient later developed persistent liquid stools, abdominal pain, intermittent fever and biochemical evidence of systemic inflammation.

## 4. Timeline

**Figure 1:**
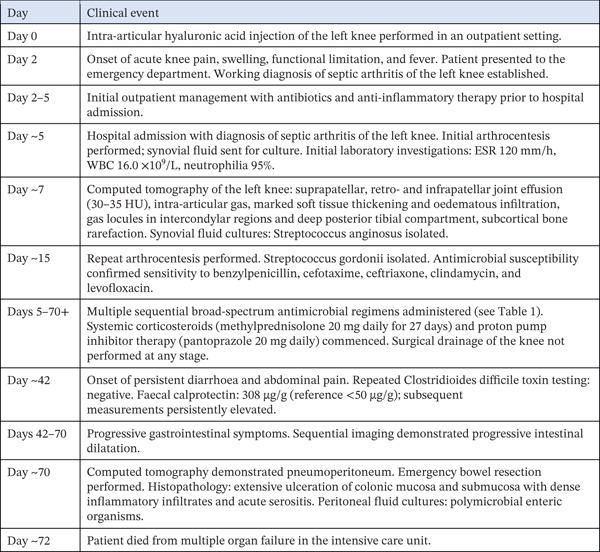
Timeline of principal clinical events. Structured tabular timeline depicting the sequence of key events from intra‐articular injection (Day 0) to death (approximately Day 72).

The clinical course extended over approximately 70 days. A structured timeline of the principal clinical events is presented in Figure 1.

Following intra‐articular hyaluronic acid injection on Day 0, symptoms of joint infection appeared on Day 2, prompting presentation to the emergency department. The patient was admitted with a diagnosis of septic arthritis of the left knee. Two days after hospital admission, a computed tomography scan of the left knee was performed. Imaging demonstrated a suprapatellar, retropatellar and infrapatellar joint effusion with fluid density values of approximately 30–35 Hounsfield units, consistent with a serohaematic effusion, associated with the presence of intra‐articular gas. Marked soft tissue thickening and oedematous infiltration of the surrounding musculature were noted in the anterolateral and posterolateral compartments of the distal left thigh, knee and proximal third of the left leg. Additional gas locules were identified in the anterior and posterior intercondylar regions, within the femorotibial joint space and in the deep posterior tibial muscle compartment. Areas of subcortical bone rarefaction with sclerotic margins were observed in the posterior paramedian distal femoral metaphysis and the proximal tibial pole.

Initial laboratory investigations revealed a markedly elevated erythrocyte sedimentation rate of 120 mm/h (reference range: 1–5 mm/h), leukocytosis with a white blood cell count of 16.0 × 10^9^/L (reference range: 4–11 × 10^9^/L) and neutrophilia of 95%. Blood cultures were negative for Gram‐positive and Gram‐negative bacteria as well as fungi.

The patient was admitted on 2 October 2024 and remained hospitalised until her death on 22 December 2024.

## 5. Diagnostic Assessment

### 5.1. Synovial Fluid Analysis

Synovial fluid analysis was performed on two occasions during the clinical course. The initial arthrocentesis was performed at the time of hospital admission (approximately Day 5 from injection). Synovial fluid analysis revealed the presence of numerous red blood cells and white blood cells. Microbiological cultures from this initial sample identified *Streptococcus anginosus*, an organism recognised for its propensity to cause deep‐seated and disseminated infections [13]. No quantitative synovial fluid leukocyte count, differential cell count or direct Gram‐staining result was recorded in the available clinical documentation. The absence of these data is a notable limitation, as quantitative white cell counts (with thresholds commonly cited at > 50,000 cells/*μ*L with > 90% neutrophils) and Gram‐stain results are considered standard components of diagnostic assessment for septic arthritis [6, 12]. The unavailability of these results may have contributed to uncertainty in clinical decision‐making regarding the timing and modality of further intervention.

A repeat arthrocentesis was performed approximately 10 days after hospital admission (approximately Day 15 from injection). This sample yielded growth of *Streptococcus gordonii*, a Gram‐positive, alpha‐haemolytic bacterium belonging to the *Streptococcus mitis* group (viridans streptococci). The isolation of a second streptococcal species on repeat aspiration raises the possibility of polymicrobial infection or, alternatively, secondary colonisation in the context of an inadequately drained joint.

Antimicrobial susceptibility testing demonstrated sensitivity to benzylpenicillin, cefotaxime, ceftriaxone, clindamycin and levofloxacin. Detailed antibiotic minimum inhibitory concentration data were not available in the accessible clinical records. Serial blood cultures obtained during hospitalisation were negative for Gram‐positive and Gram‐negative bacteria as well as fungi.

No further synovial fluid aspirations were performed after approximately Day 15. Consequently, no repeat microbiological data were available to guide subsequent antimicrobial therapy, and the decision to escalate or modify antibiotics was made on largely empirical grounds. The absence of serial synovial sampling is a significant limitation: Ongoing culture and sensitivity data would ordinarily inform targeted therapy and provide objective evidence of treatment response.

### 5.2. Gastrointestinal Assessment

With respect to gastrointestinal assessment, repeated testing for *Clostridioides difficile* toxins was consistently negative, despite the recognised association between prolonged antibiotic exposure, acid‐suppressive therapy and *C. difficile* infection [14]. Faecal calprotectin testing demonstrated persistently elevated levels, consistent with active intestinal inflammation. An initial faecal calprotectin measurement was 308 *μ*g/g (reference value: < 50 *μ*g/g), and two subsequent measurements confirmed persistently elevated values. The methodology used and the precise timing of repeat testing were not specified in the available documentation.

### 5.3. Imaging

**Figure 2:**
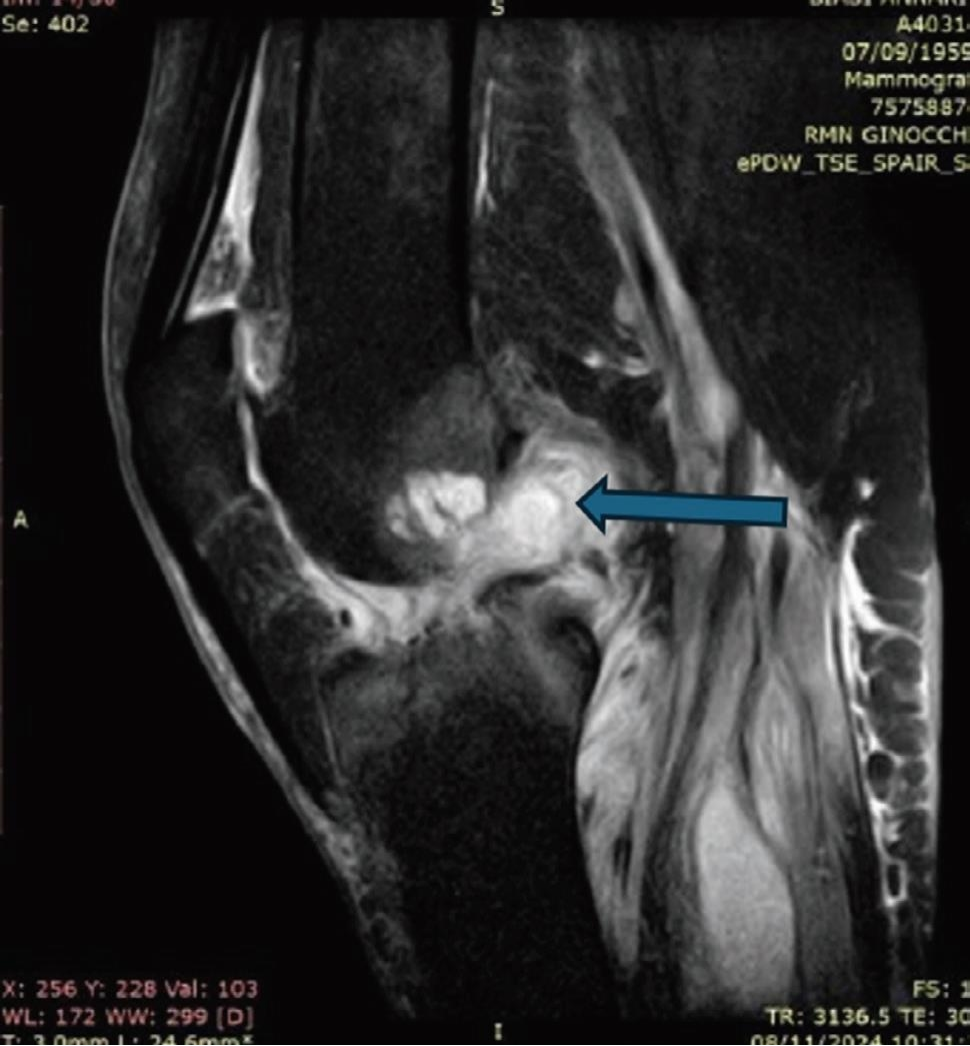
Computed tomography of the left knee, performed approximately Day 7. Annotated axial demonstrating joint effusion, intra‐articular gas, periarticular soft tissue oedema and early subcortical bone changes. Arrows indicate principal findings.

**Figure 3:**
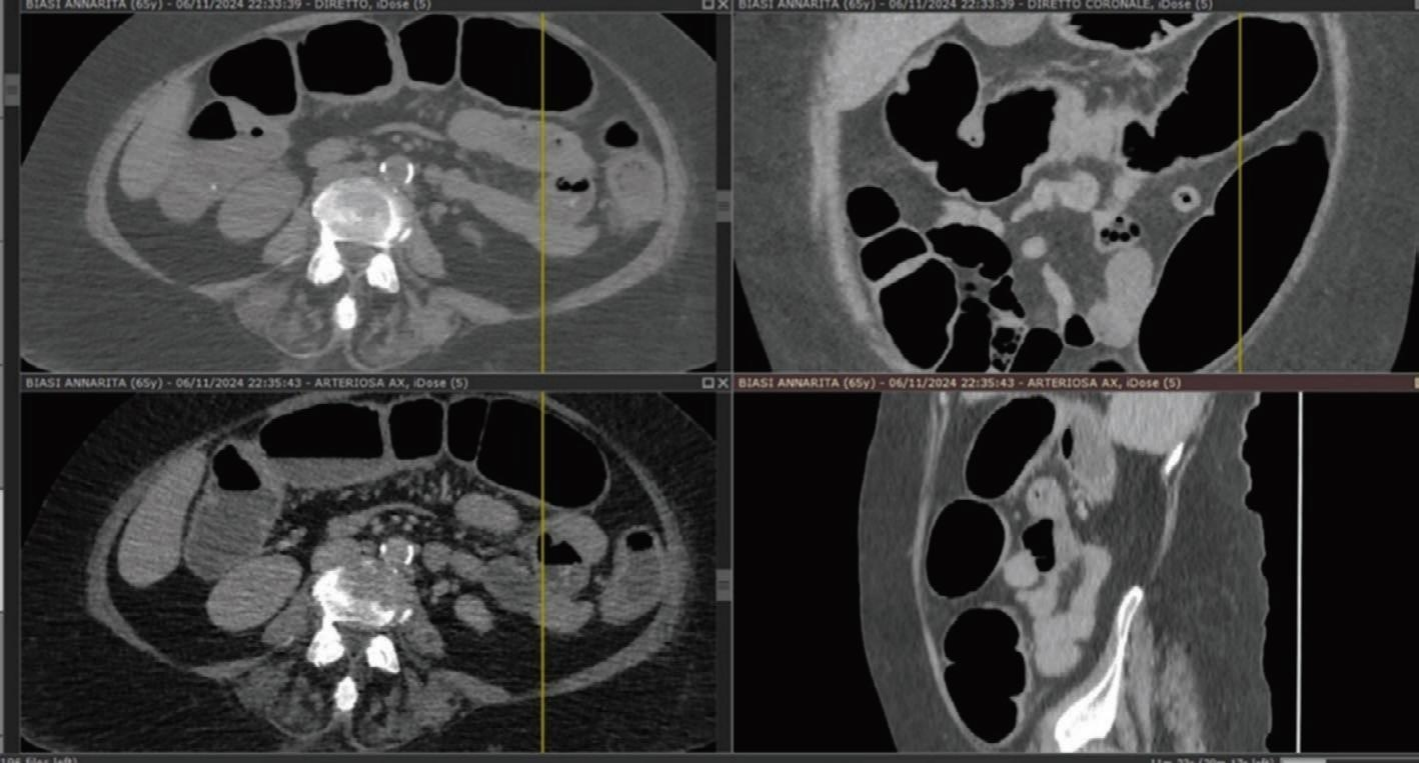
Computed tomography of the abdomen (coronal and axial projections) demonstrating mixed distention of sigmoid colon, transverse colon and rectum with air‐fluid levels. Distended and congested ileal loops with air‐fluid levels are also evident.

**Figure 4:**
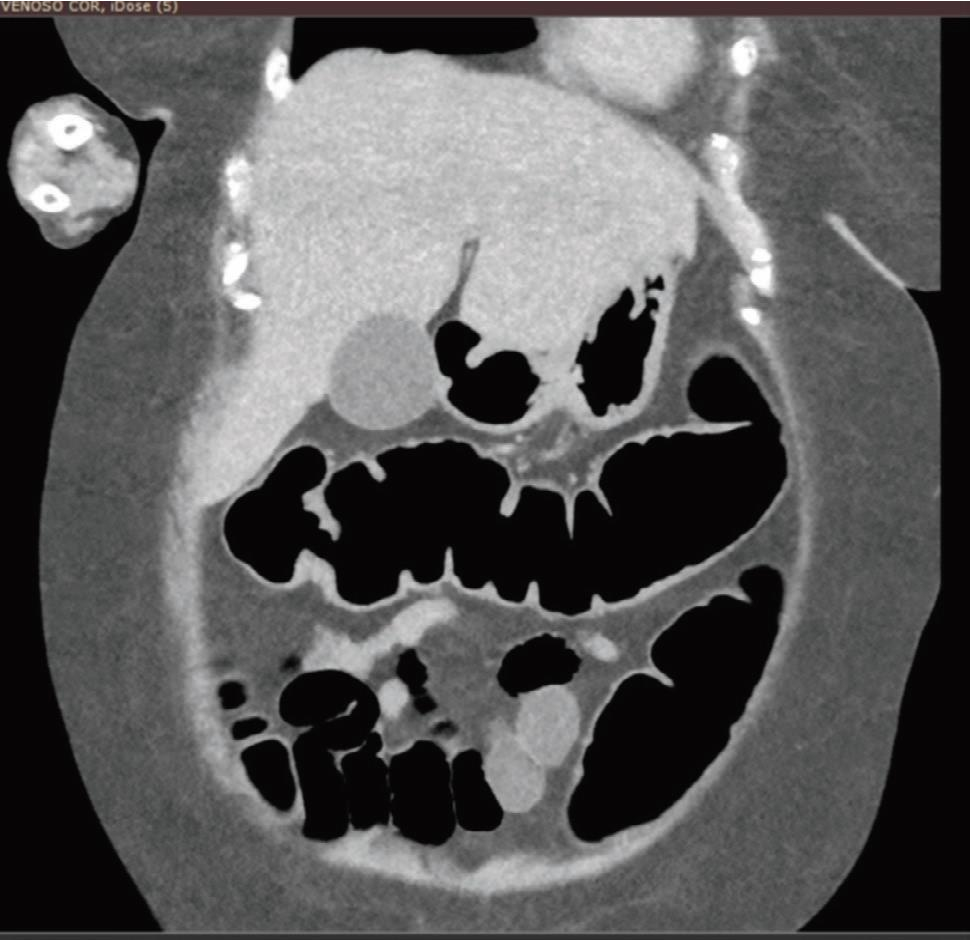
Computed tomography of the abdomen (coronal projection) demonstrating extensive bowel distention.

Computed tomography of the left knee (performed approximately Day 7) demonstrated findings consistent with a septic joint, including joint effusion with serohaematic density, intra‐articular gas, periarticular soft tissue oedema and inflammatory infiltration and early subcortical bone changes. These imaging findings are illustrated in the CT images presented in Figure 2 (see image panel). Subsequent abdominal computed tomography (performed during Weeks 6–10 of hospitalisation) demonstrated a progressive intestinal dilatation and, at approximately Day 70, pneumoperitoneum (Figures 3 and 4).

## 6. Therapeutic Intervention

During hospitalisation, the patient received multiple sequential broad‐spectrum antimicrobial regimens. The agents administered, together with available information regarding dose, route, timing and rationale for changes, are summarised in Table 1. Adjunctive pharmacological therapy, including systemic corticosteroids and acid‐suppressive treatment, is also listed.

**Table 1:** Summary of antimicrobial and adjunctive pharmacological therapy.

Drug	Dose	Route	Start	Duration	Indication/reason for Change	Susceptibility/notes
***Antimicrobial agents***						
Empirical oral antibiotics	Not specified	Oral	Days 2–5 (approx.)	3 days (approx.)	Empirical outpatient management prior to admission	N/A (preadmission)
Piperacillin–tazobactam	Not documented	Intravenous	Commenced on admission (approx. Day 5)	Duration not documented	Empirical broad‐spectrum coverage for septic arthritis	*S. anginosus* susceptible to beta‐lactams
Vancomycin	Not documented	Intravenous	Commenced during admission	Duration not documented	Escalation for suspected resistant organisms or persistent infection	Not specified
Clindamycin	Not documented	Not documented	Commenced during admission	Duration not documented	Additional Gram‐positive coverage	*S. anginosus* and *S. gordonii* susceptible
Teicoplanin	Not documented	Intravenous	Commenced during admission	Duration not documented	Glycopeptide alternative/escalation	Not specified
Metronidazole	Not documented	Not documented	Commenced during admission	Duration not documented	Anaerobic coverage; possible empirical *C. difficile* consideration	Not specified
Tigecycline	Not documented	Intravenous	Commenced during admission	Duration not documented	Broad‐spectrum escalation	Known GI adverse effect profile
Daptomycin	Not documented	Intravenous	Commenced during admission	Duration not documented	Late escalation for resistant Gram‐positive coverage	Not specified
***Adjunctive pharmacological therapy***						
Methylprednisolone (Urbason)	20 mg daily equivalent	IM and IV	During admission	27 days total	Not explicitly documented; presumed anti‐inflammatory	Immunosuppressive; impairs mucosal immune defences
Pantoprazole	20 mg daily	Oral	During admission	Duration not documented	Gastric protection during corticosteroid therapy	Associated with increased *C. difficile* risk and dysbiosis [14]

As shown in Table 1, detailed documentation regarding the dosage, route of administration and exact duration of each individual antibiotic regimen was not consistently available in the clinical records. The choice and modification of antibiotic therapy were largely empirical. Following the two initial arthrocentesis, repeated synovial fluid aspirations were not performed, and serial blood cultures remained negative for circulating bacteria; therefore, ongoing microbiological guidance for targeted antimicrobial adjustment was limited.

The patient received methylprednisolone (Urbason) administered intramuscularly and intravenously at a dose equivalent to 20 mg daily for a total duration of 27 days. This was accompanied by proton pump inhibitor therapy with pantoprazole 20 mg orally once daily. The specific clinical indications prompting initiation and continuation of corticosteroid therapy were not explicitly documented, nor were the criteria guiding its duration.

### 6.1. Adverse Events

Approximately 37 days after the initiation of antimicrobial therapy, the patient developed gastrointestinal symptoms, including persistent diarrhoea and abdominal pain. These symptoms arose in the context of cumulative exposure to at least seven distinct antimicrobial agents administered over several weeks, concurrent systemic corticosteroid therapy (which may have suppressed clinical signs of deterioration and impaired mucosal immune defences) and daily proton pump inhibitor use, which has been independently associated with increased susceptibility to enteric complications including dysbiosis‐related inflammation [11, 14].

Despite persistently elevated faecal calprotectin values (initial measurement of 308 *μ*g/g, with subsequent measurements remaining elevated) and ongoing clinical deterioration, the gastrointestinal symptoms were not investigated by endoscopy or stool culture beyond repeated negative testing for *C. difficile* toxins. Sequential imaging demonstrated a progressive intestinal dilatation over Weeks 6–10 of hospitalisation. At approximately Day 70 after admission, pneumoperitoneum was identified on computed tomography, prompting emergency bowel resection.

The prolonged duration of antimicrobial therapy and the failure to achieve source control of the primary joint infection were, in the view of the authors, contributing factors to the cumulative antimicrobial burden. Had surgical drainage been performed in a timely manner, the duration and breadth of antibiotic exposure might reasonably have been reduced, potentially mitigating the risk of secondary gastrointestinal complications. No specific laboratory drug toxicities (e.g., nephrotoxicity from vancomycin and hepatotoxicity) were documented in the available clinical records, although the absence of such documentation does not exclude their occurrence.

### 6.2. Operative Decision‐Making

Despite persistent clinical and microbiological evidence of septic arthritis, surgical drainage of the affected knee joint was not performed at any stage during the patient′s hospitalisation. The rationale for the absence of joint drainage was not documented in the available medical records.

Current consensus in the management of native joint septic arthritis holds that surgical source control, whether by serial aspiration, arthroscopic lavage or open arthrotomy, constitutes a core component of treatment alongside appropriate antimicrobial therapy [5, 6, 15]. The British Society for Rheumatology and the Sanford Guide to Antimicrobial Therapy both recommend urgent joint drainage as part of first‐line management, with repeated aspiration or surgical washout indicated in the event of persistent effusion or failure of initial aspiration to resolve infection. A recent systematic review and network meta‐analysis by Ramirez‐Acosta et al. (2025) comparing repeated joint aspiration, arthroscopic lavage and open arthrotomy in adult native knee septic arthritis found that arthroscopic lavage was associated with favourable outcomes in terms of infection eradication and functional recovery, and was considered a safe and effective intervention [5]. Similarly, Liang et al. (2022) reported that both arthroscopy and arthrotomy achieved comparable rates of infection eradication in native knee septic arthritis [15].

In this case, the CT findings at Day 7 (intra‐articular gas, serohaematic effusion, periarticular soft tissue changes and early subcortical bone rarefaction) in conjunction with positive synovial fluid cultures for *S. anginosus* would, in the view of the authors, have constituted a clear indication for surgical drainage. The *S. anginosus* group is well recognised for its capacity to form localised collections and deep‐seated infections [13], making adequate source control particularly relevant in infections caused by this organism.

The decision not to perform surgical drainage in this case is not explained in the available records and represents a departure from accepted standards of care. The authors acknowledge that there may have been clinical considerations, such as patient preference, operative risk assessment or institutional factors, that informed this decision but were not captured in the documentation available for review.

In retrospect, the authors consider that early arthroscopic lavage or, at minimum, serial joint aspiration should have been performed within the first days of admission. Prompt source control would have served several purposes: direct reduction of the bacterial burden within the joint, provision of repeat microbiological samples to guide targeted antimicrobial therapy and limitation of the total duration and breadth of empirical antibiotic exposure. The failure to achieve source control set in motion a cascade of prolonged antimicrobial therapy, progressive systemic inflammation and ultimately, the development of the secondary gastrointestinal complications described above.

## 7. Follow‐Up and Outcomes

Despite prolonged antimicrobial therapy, the joint infection persisted clinically. Gastrointestinal symptoms progressed over time, culminating in intestinal perforation. Following emergency bowel resection, the patient was admitted to the intensive care unit and subsequently died from multiple organ failure.

### 7.1. Histopathological and Microbiological Findings

Histopathological examination of the resected colon demonstrated extensive ulceration of the mucosa and submucosa with dense inflammatory infiltrates and acute serositis. Such findings are compatible with severe inflammatory or ischaemic injury but are not specific for a dysbiosis‐driven mechanism.

Peritoneal fluid cultures grew multiple enteric organisms, consistent with intestinal perforation.

## 8. Discussion

This case report describes a prolonged and fatal clinical course following septic arthritis of the knee after intra‐articular hyaluronic acid injection, a complication that, although rare, has been documented in multiple retrospective series [1–6]. The discussion that follows is structured around three principal orthopaedic lessons that emerge from this case, with the gastrointestinal complications considered as secondary consequences that inform the overall learning.

### 8.1. Lesson 1: The Consequences of Inadequate Source Control in Native Joint Septic Arthritis

The most significant management concern in this case is the absence of documented joint drainage at any stage of the patient′s treatment. Source control is a cornerstone of septic arthritis management [5, 6, 15]. Failure to achieve source control in a septic native joint may lead to persistence of the infectious focus within the joint, ongoing local tissue destruction and a state of chronic low‐grade or intermittent systemic inflammation. In this case, the absence of surgical drainage was associated with persistence of positive synovial fluid cultures on repeat aspiration (with isolation of a second streptococcal species at Day 15), failure of clinical improvement despite multiple antibiotic changes, a prolonged hospitalisation of approximately 70 days and escalating broad‐spectrum antimicrobial therapy, the cumulative burden of which was, in the authors′ assessment, a significant contributing factor to the secondary gastrointestinal complications that ultimately proved fatal.

Large observational cohorts have consistently demonstrated that outcomes in septic arthritis are adversely affected by delays in source control, even when antimicrobial therapy is initiated promptly [4, 6, 12]. The present case reinforces the principle that antimicrobial therapy alone, without adequate surgical management, is insufficient to treat native joint septic arthritis and may result in progressive harm.

### 8.2. Lesson 2: Arthroscopic Lavage as a Safe and Effective Intervention for Native Knee Septic Arthritis

Current evidence supports the use of arthroscopic lavage as a safe, effective and often preferable modality for the surgical management of native knee septic arthritis. The systematic review and network meta‐analysis by Ramirez‐Acosta et al. (2025) compared repeated joint aspiration, arthroscopic lavage and open arthrotomy, and concluded that arthroscopic lavage offered favourable outcomes in infection eradication with acceptable morbidity [5]. Liang et al. (2022), in a separate systematic review and meta‐analysis, found that arthroscopy and arthrotomy achieved similar rates of infection eradication in native knee septic arthritis, with arthroscopy associated with shorter hospital stays and lower complication rates [15].

In the present case, the patient′s clinical and imaging profile at the time of admission, including a confirmed diagnosis of septic arthritis with positive cultures for *S. anginosus*, imaging evidence of intra‐articular gas and periarticular soft tissue involvement and a background of diabetes mellitus, would have justified early arthroscopic lavage. The failure to perform such an intervention, or even to document the clinical reasoning behind its omission, represents a significant gap in management.

### 8.3. Lesson 3: Targeted Antimicrobial Therapy and Shorter Treatment Courses After Adequate Debridement

The antimicrobial course in this case was characterised by the use of at least seven distinct agents administered in sequence over many weeks, the majority selected on empirical rather than targeted grounds. This pattern is consistent with what may be expected when source control is not achieved: In the absence of surgical reduction of the infectious burden, antibiotics alone are unlikely to produce clearance, leading to serial escalation and prolonged courses.

Had adequate surgical debridement been performed early in the clinical course, the management could reasonably have followed a more targeted approach. With source control achieved through arthroscopic lavage or arthrotomy, and with repeat intraoperative cultures to confirm the organism and its susceptibility profile, antimicrobial therapy could have been narrowed to a single targeted agent (in this case, a beta‐lactam such as ceftriaxone, given the confirmed susceptibility of both isolates). Contemporary guidance and emerging evidence increasingly support shorter courses of antibiotic therapy following adequate surgical source control in bone and joint infections, with regimens of 2–4 weeks of intravenous therapy considered sufficient in many settings where debridement has been satisfactorily performed [6].

A shorter, more targeted antimicrobial course would have reduced cumulative drug exposure and, in turn, may have lowered the risk of the antibiotic‐associated complications observed in this patient. The associations between prolonged antibiotic use and intestinal dysbiosis, inflammatory bowel disease and antibiotic‐associated colitis are well documented in the literature [7–9, 16–19]. Although a direct causal link between the antimicrobial therapy and the colonic perforation in this case cannot be established, the temporal association is noteworthy, and the cumulative burden of multiple broad‐spectrum agents over many weeks represents a recognised risk factor for the type of gastrointestinal injury described.

### 8.4. Gastrointestinal Complications as a Secondary Consequence

The gastrointestinal manifestations in this case evolved over weeks. Although *C. difficile* testing was repeatedly negative, reliance on a single pathogen‐focused diagnostic approach may provide false reassurance. Antibiotic‐associated colitis, ischaemic colitis, medication‐induced mucosal injury and dysbiosis‐related inflammation remain plausible alternative diagnoses [19–22]. The absence of stool cultures, endoscopic assessment and targeted gastrointestinal investigation represents a significant diagnostic limitation.

Histological findings confirmed severe inflammatory injury but could not establish a specific aetiology. Similarly, polymicrobial peritoneal cultures reflect the consequences of perforation rather than its cause. *S. anginosus* group organisms are recognised for their invasive potential and may contribute to systemic complications, although direct colonic involvement could not be demonstrated in this case [13].

The gastrointestinal complications are reported here not as a primary focus of the case, but as an illustration of the downstream systemic consequences that may follow from inadequate orthopaedic management of a septic joint. They underscore the principle that failure to achieve source control at the primary site of infection may have cascading effects on other organ systems, mediated in part by the therapeutic burden required to manage an uncontrolled infection.

## 9. Limitations

This report is subject to several limitations inherent to retrospective case analysis. Detailed documentation regarding the rationale for clinical decisions (particularly the omission of joint drainage), the timing and dosing of medications and the results of certain diagnostic investigations was not consistently available. The absence of quantitative synovial fluid analysis, serial aspirations after Day 15, stool cultures and endoscopic assessment limits the conclusions that can be drawn regarding the mechanisms underlying the gastrointestinal deterioration. As a single case report, causality cannot be established, and the observations described should be interpreted as hypothesis‐generating rather than definitive.

## Author Contributions

Fabio Massimo Abenavoli, MD, was involved in conceiving the idea, writing the initial draft and designing the methodology. Omar Tujjar, MD, conceptualised the study and provided a critical review. Andrea Alfonso, MD, provided the final approval of the manuscript.

## Funding

No funding was received for this manuscript.

## Ethics Statement

This report describes a single clinical case and does not constitute interventional research or a clinical trial. Under the institutional policy of the authors′ affiliated hospitals, individual case reports based on retrospective review of clinical data with appropriate informed consent do not require formal institutional review board (IRB) or research ethics committee approval. The study was conducted in accordance with the principles of the Declaration of Helsinki. Written informed consent for publication was obtained from the patient′s next of kin as described above.

## Consent

Written informed consent for publication of this case report, including all clinical details and accompanying images, was obtained from the patient′s next of kin following their review of the final manuscript text and all figures. The consenting party was informed of the risk of online reidentification that may exist despite the removal of direct identifiers and consented to publication with this understanding. Nonessential identifying details of the patient and medical history have been omitted to maintain anonymity to the greatest extent possible. The original signed consent form is retained by the corresponding author and is available for inspection by the journal upon request, in accordance with editorial policy.

## Conflicts of Interest

The authors declare no conflicts of interest.

## Supporting information


**Supporting Information** Additional supporting information can be found online in the Supporting Information section. CARE: Case Report checklist with page references.

## Data Availability

Data sharing is not applicable to this article as no datasets were generated or analysed during the current study.
