# Supplementary material for: Neglected Joint Infection Occurring Following Intra‐Articular Injection and Colon Perforation: A Case Report
Source: Case Rep Orthop. 2026 Apr 25;2026:1431586. doi: 10.1155/cro/1431586 (PMC13110352; doi:10.1155/cro/1431586)
Supplement: Supplementary file 1 — Supporting Information Additional supporting information can be found online in the Supporting Information section. CARE: Case Report checklist with page references. [file CRO-2026-1431586-s001.docx]

# Appendix: CARE Checklist

***CARE Case Report Checklist with page references.***

| **CARE Checklist Item** | **Description** | **Page/Section** |
| --- | --- | --- |
| **Title** | The area of focus and "case report" identified in the title | 1 |
| **Key Words** | 2 to 5 key words that identify topics in this case report | 1 |
| **Abstract** | Introduction: What is unique and why is it important? | 2 |
| **Abstract** | Case Presentation: The main symptoms, clinical findings, diagnoses, interventions, and outcomes | 2 |
| **Abstract** | Conclusion: What are the main take-away lessons? | 2 |
| **Introduction** | One or two paragraphs summarising why this case is unique with medical literature references | 3 |
| **Patient Information** | De-identified demographic and other patient information | 4 |
| **Patient Information** | Main concerns and symptoms of the patient | 4 |
| **Patient Information** | Medical, family, and psychosocial history including genetic information | 4 |
| **Patient Information** | Relevant past interventions and their outcomes | 4 |
| **Clinical Findings** | Describe the relevant physical examination (PE) findings | 5 |
| **Timeline** | Depict important dates and times in this case as a figure | 6–7 |
| **Diagnostic Assessment** | Diagnostic methods (PE, laboratory testing, imaging, etc.) | 8–9 |
| **Diagnostic Assessment** | Diagnostic challenges | 8–9 |
| **Diagnostic Assessment** | Diagnostic reasoning including other diagnoses considered | 8–9 |
| **Diagnostic Assessment** | Prognostic characteristics where applicable | 8–9 |
| **Therapeutic Intervention** | Types of intervention (pharmacologic, surgical, preventive) | 10–12 |
| **Therapeutic Intervention** | Administration of intervention (dosing, strength, duration) | 10–12 |
| **Therapeutic Intervention** | Changes in the interventions with rationale | 10–12 |
| **Follow-up and Outcomes** | Clinician-assessed and patient-assessed outcomes when appropriate | 12 |
| **Follow-up and Outcomes** | Important follow-up diagnostic and other test results | 12 |
| **Follow-up and Outcomes** | Intervention adherence and tolerability | 12 |
| **Follow-up and Outcomes** | Adverse and unanticipated events | 11–12 |
| **Discussion** | Strengths and limitations in the management of this case | 13–15 |
| **Discussion** | Discussion of the relevant medical literature | 13–15 |
| **Discussion** | The rationale for conclusions | 13–15 |
| **Discussion** | The primary take-away lessons from this case report | 13–15 |
| **Patient Perspective** | The patient or next of kin shared their perspective on the treatment(s) | N/A (deceased patient; consent obtained from next of kin) |
| **Informed Consent** | Did the patient give informed consent? Please provide if requested | 15–16 |
